# Clinical and functional characterization of a novel *STUB1* frameshift mutation in autosomal dominant spinocerebellar ataxia type 48 (SCA48)

**DOI:** 10.1186/s12929-021-00763-1

**Published:** 2021-09-26

**Authors:** Huan-Yun Chen, Chia-Lang Hsu, Han-Yi Lin, Yung-Feng Lin, Shih-Feng Tsai, Yu-Jung Ho, Ye-Ru Li, Jin-Wu Tsai, Shu-Chun Teng, Chin-Hsien Lin

**Affiliations:** 1 Department of Microbiology, College of Medicine, National Taiwan University, No. 1, Section 1, Jen-Ai Road, Taipei, 10051 Taiwan; 2 Department of Medical Research, National Taiwan University Hospital, Taipei, Taiwan; 3 Department of Neurology, National Taiwan University Hospital, Number 7, Chung-Shan South Road, Taipei, 10051 Taiwan; 4 Department of Life Sciences and Institute of Genome Sciences, National Yang-Ming University, Taipei, Taiwan; 5 Institute of Molecular and Genomic Medicine, National Health Research Institutes, Zhunan, Taiwan; 6 Institute of Brain Science, College of Medicine, National Yang Ming Chiao Tung University, Taipei, 112 Taiwan; 7 Brain Research Center, National Yang Ming Chiao Tung University, Taipei, 112 Taiwan; 8 Center of Precision Medicine, National Taiwan University, Taipei, Taiwan

**Keywords:** Spinocerebellar ataxia type 48, Ataxia, CHIP, STUB1, Tau, α-Synuclein

## Abstract

**Background:**

Heterozygous pathogenic variants in *STUB1* are implicated in autosomal dominant spinocerebellar ataxia type 48 (SCA48), which is a rare familial ataxia disorder. We investigated the clinical, genetic and functional characteristics of *STUB1* mutations identified from a Taiwanese ataxia cohort.

**Methods:**

We performed whole genome sequencing in a genetically undiagnosed family with an autosomal dominant ataxia syndrome. Further Sanger sequencing of all exons and intron–exon boundary junctions of *STUB1* in 249 unrelated patients with cerebellar ataxia was performed. The pathogenicity of the identified novel *STUB1* variant was investigated.

**Results:**

We identified a novel heterozygous frameshift variant, c.832del (p.Glu278fs), in *STUB1* in two patients from the same family. This rare mutation is located in the U-box of the carboxyl terminus of the Hsc70-interacting protein (CHIP) protein, which is encoded by *STUB1*. Further in vitro experiments demonstrated that this novel heterozygous *STUB1* frameshift variant impairs the CHIP protein’s activity and its interaction with the E2 ubiquitin ligase, UbE2D1, leading to neuronal accumulation of tau and α-synuclein, caspase-3 activation, and promoting cellular apoptosis through a dominant-negative pathogenic effect. The in vivo study revealed the influence of the CHIP expression level on the differentiation and migration of cerebellar granule neuron progenitors during cerebellar development.

**Conclusions:**

Our findings provide clinical, genetic, and a mechanistic insight linking the novel heterozygous *STUB1* frameshift mutation at the highly conserved U-box domain of CHIP as the cause of autosomal dominant SCA48. Our results further stress the importance of CHIP activity in neuronal protein homeostasis and cerebellar functions.

**Supplementary Information:**

The online version contains supplementary material available at 10.1186/s12929-021-00763-1.

## Background

C-terminus of HSC70-interacting protein (CHIP), encoded by the gene *STUB1*, functions as both a molecular co-chaperone and a ubiquitin E3 ligase, which plays a pivotal role in regulating cellular protein homeostasis [1]. CHIP contains three domains, including an N-terminal three tetratricopeptide repeat (TPR) domain, a highly charged middle coiled-coil domain, and a carboxyl-terminal U-box domain [1, 2]. CHIP acts as a co-chaperone of heat shock protein 70 (HSC70)/HSP70 and HSP90 through the TPR domain, and also acts as an E3 ligase, its U-box domain tagging chaperone-bound substrates with ubiquitin. Emerging evidence indicates that CHIP is implicated in regulating multiple fundamental cellular processes, including refolding and degradation of misfolded proteins, autophagy, immunity, and necroptosis [3–5]. Although CHIP is ubiquitously expressed, its expression is elevated in tissues with high metabolic rates, especially brain tissues [2]. The degradation of those proteins or organelles via the ubiquitin–proteasome system (UPS) plays a crucial role in protein quality control and sustains proper cellular homeostasis, particularly important in neurons [6, 7].

The spinocerebellar ataxias (SCAs) comprise a heterogeneous group of disorders characterized by progressive cerebellar ataxia. Mutations in *STUB1* in homozygous or compound heterozygous states were originally reported to cause autosomal recessive spinocerebellar ataxia type 16 (SCAR16), with widespread neurodegeneration manifesting as an ataxic gait disorder combined with a wide spectrum of phenotypes, including epilepsy, cognitive decline, chorea, pyramidal sign, sensory polyneuropathy, and hypogonadism, also known as Gordon Holmes syndrome [8, 9]. The in vitro studies have shown that biallelic mutations in *STUB1*, especially those located in the U-box domain, induce a loss of CHIP function by decreasing the interaction with chaperones and diminishing the ubiquitin–proteasome system, thereby promoting the formation of misfolded proteins [8–10]. The homozygous *STUB1* knockout mice displayed ataxia and cognitive impairment, mimicking patients with SCAR16. Histological examinations revealed a neuronal loss throughout the cerebellum, especially in the Purkinje cells, compared with those in wild-type mice, suggesting a vital role of CHIP in maintaining cerebellar development and function [8]. Notably, single heterozygous mutations, mostly frameshift mutations, in *STUB1* have recently been described as a cause of autosomal dominant spinocerebellar ataxia type 48 (SCA48), with later disease onset, milder disease presentation with the features of ataxia, cognitive decline, and mood disorders [11]. The same phenomena were also observed in both SCAR15 and SCA5, due to recessive and dominant mutations in *SPTBN2*, respectively [12, 13]. Since patients with SCA48 present similar symptoms as those with SCAR16, CHIP must be a critical element in maintaining the cerebellar function, but the detailed mechanism is not completely understood.

Recent advances in genetic studies have identified more than 40 genes causing distinct subtypes of SCA [14]. We and other groups have previously described the clinical features of Taiwanese patients with the most common genetic causes of SCA [15, 16]. To extend our knowledge of the genetic architecture and pathophysiology of SCA in our population, we performed whole-genome sequencing (WGS) and comparative analysis in a genetically undiagnosed multiplex family with SCA. Trinucleotide repeat expansion mutations in the most prevalent SCA1, 2, 3, 6, 17, and dentatorubral-pallidoluysian atrophy (DRPLA) were excluded. We further investigated the mutation frequency of *STUB1* in a cohort of ataxia patients without a known molecular diagnosis. The functional effect of the identified novel heterozygous *STUB1* variant was subsequently examined in vitro in neuronal cell lines and in vivo in a mouse model to assess its neuronal pathogenicity.

## Methods

### Participants and clinical examination

**Fig. 1:**
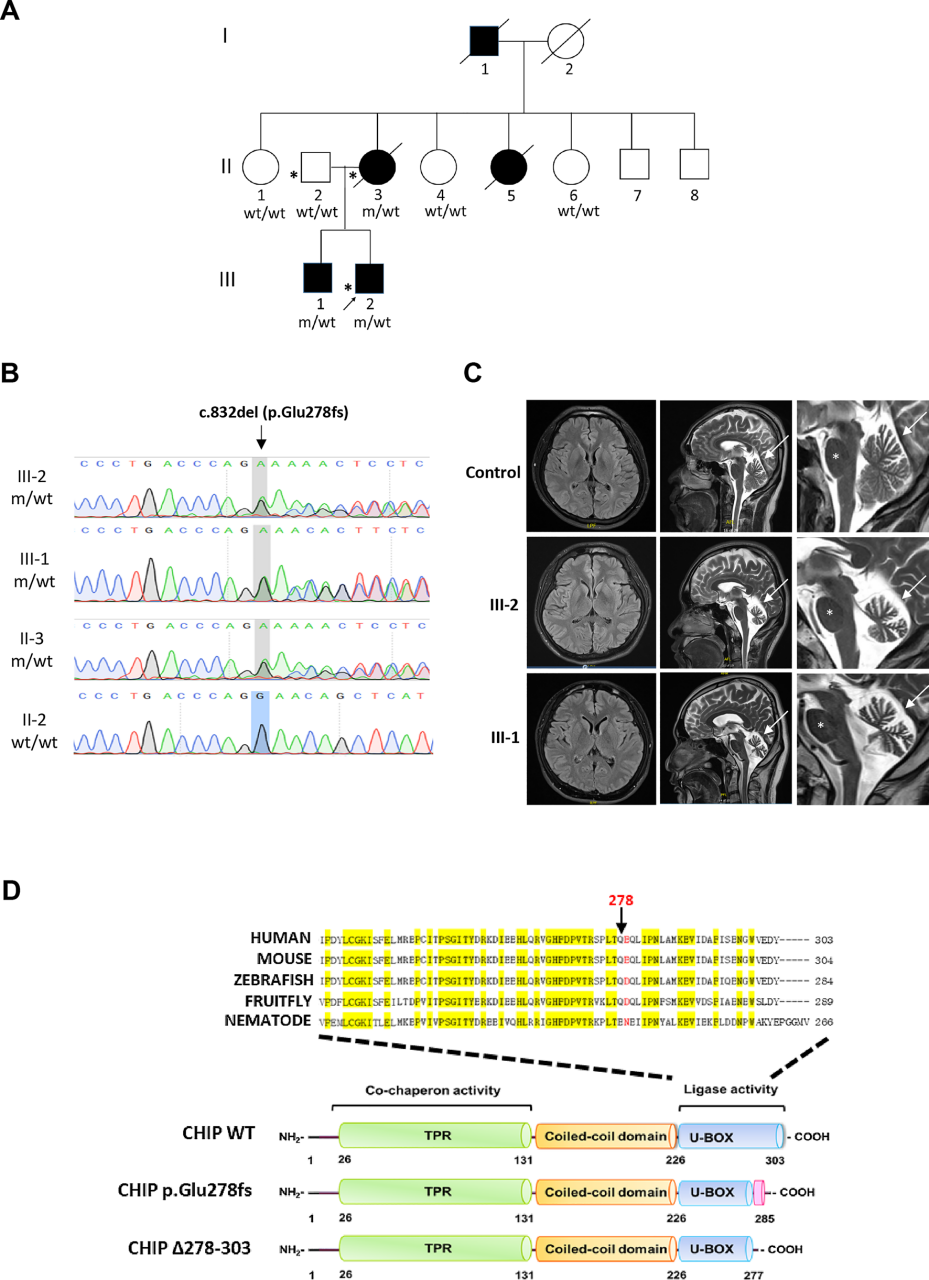
Pedigree, genetic and brain MRI features of the index SCA48 family with the novel *STUB1* frameshift mutation. **A** Pedigree of the index family with the heterozygous rare frameshift variant, c.832del (p.Glu278fs), in the *STUB1* gene. m/wt = heterozygous carriers of the *STUB1* mutation; wt/wt = non-carriers; open symbols = unaffected; filled symbols = affected; symbol with a diagonal line = deceased; arrow = proband. Asterisks indicate family members whose whole genomes were sequenced. **B** Sanger sequencing traces confirming the c.832del (p.Glu278fs) variant in *STUB1* identified in the proband and affected members of the index family. **C** Brain MRI scans show cerebellar atrophy (arrows) with preserved pons (asterisk) in patients III:1 and III:2 of the index family, and an age- and gender-matched healthy control participant. **D** CHIP comprises three functional domains: TPR, coiled-coil, and U-Box. The protein structure shows that the p.Glu278fs variant amino acid residue (red) is located in the U-Box domain of CHIP. The CHIP Δ278–303 mutation results in a truncated protein without the C-terminal 22 aa of the U-box domain (278–303). A sequence alignment (top) demonstrates the evolutionary conservation of E278 in the U-Box domain of CHIP proteins across the indicated species. Identical residues are labeled in yellow

For the index family with autosomal dominant cerebellar ataxia syndrome (Fig. 1A), whole blood was collected from two affected individuals and one asymptomatic member as a trio for WGS analyses. Another 249 independent patients with cerebellar ataxia lacking a molecular diagnosis were recruited from the movement disorder clinic of National Taiwan University Hospital. The diagnosis of SCA was made according to the Harding diagnostic criteria [17]. Autosomal dominant inheritance was defined by the presence of at least one other affected individual among parents or children of the index case in 32 probands. Ten families were suggestive of a recessive model of inheritance, and 207 were sporadic cases. The study protocol was approved by the institutional review board of National Taiwan University Hospital. All participants signed written informed consent.

### Genetic analysis of known common SCA genes

Genomic DNA was isolated from 10 mL of venous blood from all participants, following a standard protocol. Trinucleotide repeat expansions at the SCA1, 2, 3, 6, 17 and DRPLA genes were excluded from all participants, following the methods described previously [15].

### Whole-genome sequencing and data analysis

WGS was performed in individuals II-2, II-3, and III-2 of the index family (Fig. 1A). Paired-end multiplex libraries were prepared, according to the manufacturer’s instructions, with an Illumina (San Diego, CA) TruSeq DNA Sample Prep Kit and enriched with the NimbleGen SeqCap EZ Human Exome Library v3.0. The NimbleGen kit targets 64 Mb, corresponding to 30,000 genes. Libraries were loaded into Illumina flow cells for cluster generation before producing 100-base read pairs on a HiSeq2000 instrument, following the Illumina protocol. Base-calling and quality control were done with the Illumina RTA sequence analysis pipeline according to the manufacturer’s instructions.

Reads were hard trimmed from the end of the read up to the first base with a quality of at least 10. Reads with at least 40 nt of length were mapped to Human Genome build hg19 using the Genome Multitool v1 Application (GEM mapper) 31 allowing up to 4 mismatches. Alignment (.bam) files containing only properly paired, uniquely mapping reads were processed using Picard tools (broadinstitute.github.io/picard/) version 1.110 to add read groups and to remove duplicates. We removed common variants in the population that had minor allele frequencies > 1% in dbSNP version 151 [18] or the Taiwan BioBank [19]. For variants in the coding region, we used PROVEAN [20], SIFT [21], and PolyPhen-2 [22] to predict the potential impact of the variant on protein structure and function, and removed the variants that do not significantly affect protein structure. We also filtered out the variants that have been reported in the ClinVar database with their types belonging to benign or likely benign. The Human Phenotype Ontology (HPO) database was used to identify candidate genes based on the patient’s ataxia phenotype and the HPO ID is HP:0001251. The ataxia candidate genes for this study including 804 genes are listed in the Additional file 1: Table S1. By comparison with the ataxia candidate gene list, we selected the variants located in the ataxia candidate genes and in the functional regions including coding region, 5’-UTR, 3’-UTR or splicing site. Finally, we performed the trio-based analysis and used the family disease history to conduct the dominant inheritance model analysis. Varsome version 10.0 tool platform (https://varsome.com/) was applied for the classification of pathogenicity based on the American College of Medical Genetics and Genomics interpretation criteria [23].

### Sanger sequencing and segregation analysis

The identified potentially pathogenic variant was ascertained by Sanger sequencing. Primer sequences and PCR conditions were described previously [11]. We had DNA available and detailed phenotypic information from seven individuals, three affected and four unaffected, who were included in the further segregation analysis (Fig. 1A).

A further screening of all exons and exon–intron boundary junctions of the *STUB1* gene using Sanger sequencing was performed on the other 249 unrelated patients with cerebellar ataxia.

### Cell culture and reagents

Human neuroblastoma SH-SY5Y and BE2-M17 cells were cultured in DMEM/F12 (44.5/44.5%) supplemented with 10% FBS and antibiotics (100 U/mL of penicillin and 100 μg/mL of streptomycin) at 37 °C with 5% CO_2_.

### Western blotting and antibodies

Cells were harvested in cell lysis buffer and proteins were separated using 12% SDS-PAGE. The target proteins were detected using an enhanced chemiluminescent reagent (GE Healthcare, USA). The antibodies used for immunoblotting were anti-CHIP (Bethyl Laboratories, USA); anti-tau (Genetex, USA); anti-UBE2D1, anti-UBE2D2 and anti-UBE2D3 (Abnova, Taiwan); anti-FLAG (Sigma-Aldrich, USA), anti-Myc (Roche, Switzerland), anti-caspase-3 (Cell Signaling Technology, USA) and anti-β-actin (Sigma-Aldrich, USA).

### Plasmids and cell transfection

pcDNA3.1-myc-STUB1 was a kind gift from Dr. Pamela J. McLean [24]. The CHIP p.Glu278fs and CHIP Δ278-303 mutations were introduced by PCR amplification using a Phusion High-Fidelity kit (Thermo Scientific, USA). The open reading frame encoding human tau was amplified from the Myc-tau plasmid [25] and cloned into the pEGFP-C1 vector [26]. The full-length CHIP, CHIP p.Glu278fs, or CHIP Δ278–303 fragments were cloned into the pCMV-Tag2B vector at the HindIII/EcoRI sites to create an N-terminal Flag tag. Cells were transfected with Lipofectamine LTX Reagent (Thermo Fisher Scientific, USA) according to the manufacturer’s instructions.

### Immunofluorescence and confocal microscopy

Cells were transfected with CHIP WT, p.Glu278fs, or CHIP Δ278–303, with or without tau-EGFP, and seeded onto glass coverslips (Marienfeld Laboratory Glassware, Germany) at 4 × 10^5^ cells/mL in 6-well plates and fixed with 4% paraformaldehyde in PBS for 20 min at room temperature. Fixed cells were washed with PBS and permeabilized with 0.1% Triton X-100 in PBS for 5 min. After washing with PBS, the coverslips were incubated with α-synuclein (Genetex, USA)-specific antibodies overnight at 4 °C. The coverslips with cells transfected with CHIP WT, p.Glu278fs, or p.Glu278 were incubated with Rhodamine Red-X-conjugated goat-anti-rabbit IgG (H + L) (Jackson ImmunoResearch, USA) overnight at 4 °C. After two washes with PBS, the coverslips were stained with DAPI for 10 min, and cells were mounted with a mounting medium (Sigma, USA). Confocal images were captured under a Zeiss LSM880 laser scanning fluorescence confocal microscope. The percentage of cells with α-synuclein aggregated foci was determined by counting at least 100 cells per strain, using Image J software.

### Co-immunoprecipitation (Co-IP)

For the E2 association assay, cells transfected with CHIP WT, CHIP p.Glu278fs, or CHIP Δ278–303 were harvested in NP40 lysis buffer (50 mM Tris–HCL pH 7.5, 150 mM NaCl, 2 mM EDTA, 1 mM NA_3_VO_4_, 0.1 M NaF, 0.1% NP40, 1 mM PMSF). Cell lysates (1 mg) were incubated overnight with anti-FLAG antibodies (3.8–4.2 μg, Sigma-Aldrich, USA) at 4 °C. For the dimerization assay, cells transfected with CHIP-Myc along with CHIP WT-FLAG, CHIP p.Glu278fs-FLAG, CHIP Δ278–303-FLAG were harvested in NP40 lysis buffer. Cell lysates (1 mg) were incubated overnight with anti-FLAG antibodies (3.8–4.2 μg, Sigma-Aldrich, USA) or anti-Myc antibodies (2 μg, Roche, Switzerland) at 4 °C. Immunocomplexes were isolated with protein A-Sepharose beads saturated with 1% bovine serum albumin (BSA) by rotating for 5 h at 4 °C. After washing, bound proteins were denatured, eluted, and resolved by 12% polyacrylamide SDS-PAGE.

### Filter-trap assay

SH-SY5Y and BE2-M17 cells were transfected with a plasmid expressing CHIP WT, CHIP p.Glu278fs, or CHIP Δ278–303. For the dominant-negative test, cells were transfected with a plasmid expressing CHIP WT, CHIP p.Glu278fs, or co-transfected with both CHIP WT and CHIP p.Glu278fs plasmids. Cell pellets were collected and lysed with a buffer containing 50 mM Tris, pH 7.5, 150 mM NaCl, 2 mM EDTA, 1 mM Na_3_VO_4_, and 0.1% NP40. The samples were mixed with SDS to a final concentration of 2% and filtered through a 96-well dot blot apparatus (Bio-Rad Laboratories, USA) containing a 0.2-μm nitrocellulose membrane. The nitrocellulose membrane was then probed with the anti-α-synuclein antibody (Genetex, USA) or tau antibody (Genetex, USA). Chemiluminescence was quantified using ImageJ software.

### Bioinformatics analysis of STUB1 interactome

Identification of *STUB1*-interacting proteins and pathway enrichment analysis of these proteins was performed using Ingenuity Pathway Analysis (IPA) [27]. The *STUB1* interactome was visualized as networks using Cytoscape [28].

### Animal model and cerebellar electroporation

ICR mice were used for cerebellar electroporation. All protocols were approved by the Institutional Animal Care and Use Committee (IACUC) at National Yang Ming University. For *STUB1* knockdown, shRNA plasmids based on PLKO.1-puro backbone were purchased from Academia Sinica RNAi Core, Taiwan (*STUB1* shRNA: GAGAGTGAGCTGCATTCATAT; control scramble shRNA CCTAAGGTTAAGTCGCCCTCG). The knockdown efficiency was confirmed in SH-SY5Y neuroblastoma cells. The *STUB1* plasmid based on the pCIG2 vector was used for *STUB1* overexpression. Cerebellar electroporation was performed according to previous studies [29, 30]. Briefly, ICR mouse pups at P6 were anesthetized on ice for 2 min until they had no response to pain stimulation. The occipital skin was cut using a surgical knife to expose the skull above the cerebellum. A 26-gauge needle was used to drill a tiny hole in the skull. A 33-gauge needle was then inserted into the cerebellum for plasmid DNA delivery, and 3–6 μg of DNA was injected into the primary fissure of the cerebellum. After injection, a tweezer-type electrode connected to a square wave generator (ECM 830, Harvard Apparatus) was placed on the occipital region of the brain and provided a short current of 70 V (V), with 6 pulses of 50-ms duration at 150-ms intervals. The wound was sutured and sterilized with 70% alcohol after surgery.

### Immunohistochemistry and microscopy

The cerebellum was collected through cardiac perfusion of PBS followed by 4% paraformaldehyde (PFA). The cerebellum was sagittally sectioned using a Vibrotome (Leica) into 100-μm slices. The slices were washed with PBS and permeated by PBST (0.2% Triton X-100 in PBS) for 30 min. The slices were then incubated in blocking buffer (10% normal goat serum [NGS] + 5% BSA in PBST) for at least 1 h at room temperature. Primary antibodies (Ki67, rabbit polyclonal, 1:500, ab15580) were diluted in blocking buffer (PBST containing 5% NGS and 5% BSA) and the slices were incubated with them at 4 °C for 2 nights. They were then washed and incubated with a fluorescent secondary antibody (goat anti-rabbit 555, 1:500, Alexa Fluor 11,010) for 2 h. Finally, the slices were washed, stained with DAPI (Invitrogen) for 1 h, and mounted in Vectashield Mounting Medium on slides.

### Statistical analysis

Paired data were expressed as means ± standard errors of the mean (SEM). The Student t-test with a two-tailed distribution was used for statistical comparisons between two groups. The one-way ANOVA post hoc Tukey test was performed for comparisons among multiple groups. Statistical tests were performed using Stata (StataCorp LP, College Station, Texas) and GraphPad Prism 8.0.1 version. A two-sided *P* < 0.05 was considered significant.

### Data availability statement

Source data are provided with this paper. The datasets generated and analyzed during the current study are available from the corresponding author on reasonable request.

## Results

### Clinical and genetic characterization of a novel heterozygous frameshift mutation in *STUB1*

Clinical features and pedigree of the index three-generation autosomal-dominant cerebellar ataxia family, including three symptomatic and four asymptomatic members, were summarized in Table 1 and Fig. 1A. The median age at onset was 30 years and the median age at diagnosis was 40 years. The clinical presentation was a slowly progressive, cerebellar ataxic gait, followed by a slow saccadic eye movement, scanning speech, and limb dysmetria in all affected members. Cognitive function was affected, with the complete neuropsychological tests revealing impaired frontal shifting speed and recent memory. The mini mental state examination score (MMSE) was 28/30 in two affected members (III-1 and III-2). The MMSE was 24/30 for the proband’s affected mother (II-3) at the age of 60 years. There was no rigidity, parkinsonism, chorea, or other movement disorder observed in the affected members. The proband’s mother (II-3) was wheelchair bound when examined and died from aspiration pneumonia at the age of 63 years. The brain MRI scans of the proband (III-2) and his elder brother (III-1) showed markedly global cerebellar atrophy with pons sparing (Fig. 1C).

**Table 1:** Demographic and clinical features of the index family carrying the heterozygous *STUB1* mutation

	III:1	III:2	II:1	II:2	II:3	II:4	II:5	II:6	II:7	II:8	I:1	I:2
Current age (year)	30	28	66	65	63	62	55	59	57	55	60	70
Onset age (year)	23	24	N.A	N.A	38	N.A	30	N.A	N.A	N.A	30	N.A
Age at death (year)	N.A	N.A	N.A	N.A	64	N.A	55	N.A	N.A	N.A	60	N.A
Sex	M	M	F	M	F	F	F	F	M	M	M	F
Cerebellar dysfunction related symptoms												
Ataxia	** + + + **	** + **	**−**	**−**	** + + + **	**−**	** + + + **	**−**	**−**	**−**	** + + + **	N.A
Dysarthria	** + + **	** + **	**−**	**−**	** + + **	**−**	** + + **	**−**	**−**	**−**	** + + + **	N.A
Dysphagia	** + **	**−**	**−**	**−**	** + + **	**−**	** + + **	**−**	**−**	**−**	** + + **	N.A
Hands dysmetria	** + + **	** + + **	**−**	**−**	** + + **	**−**	** + **	**−**	**−**	**−**	** + + **	N.A
Cognitive-affective symptoms												
MMSE	28	28	30	30	24	29	N.A	30	30	30	N.A	N.A
Depression	** + **	** + **	**−**	**−**	** + + **	**−**	** + + **	**−**	**−**	**−**	** + + **	**−**
Anxiety	**−**	**−**	**−**	**−**	**−**	**−**		**−**	**−**	**−**	**−**	**−**
Associated symptoms												
Polyneuropathy	**−**	**−**	**−**	**−**	**−**	**−**	**−**	**−**	**−**	**−**	N.A	N.A
Activities of daily living												
Waling disability	Crutches	Wo aid	Well	N/A	WC	Well	WC	Well	Well	Well	WC	Well
Dependency	P	I	I	I	T	I	T	I	I	I	T	I

*M* male; *F* female; *MMSE* Mini-Mental State Examination; *wo aid* without aid; *WC* wheelchair bounded; *P* partially dependent; *I* independent; *T* totally dependent

The proband (III-2) did not carry abnormal trinucleotide repeat numbers at the SCA1, 2, 3, 6, 17 and DRPLA genes. The DNA samples of the proband, the affected mother, and the unaffected father were then sent for WGS analysis. The average percentage of coverage of the WGS was at least 97.5% of the target region covered by at least 10 sequencing reads. The mapping information of the individuals II-2, II-3, and III-2 was detailed in the Additional file 1: Table S2. The filtering information of the variants identified from the proband (III-2) was detailed in the Additional file 1: Table S3. We then identified two candidate heterozygous variants, including *SETX* c.7747T > C (p.F2554L) and *STUB1* c.832del (p.Glu278fs), for the proband. Only the heterozygous *STUB1* c.832del (chr16: 732,408, p.Glu278fs) co-segregated with the disease within the index family (Fig. 1A, B). All three patients, but not the four asymptomatic family members, have a heterozygous mutation (c.832del, p.Glu278fs) in the *STUB1* gene (transcript NM_005861) (Fig. 1A). This novel single-base deletion at nucleotide 832 in these cases leads to a frameshift at the conserved glutamic acid (Glu) residue 278 which deletes the C-terminal tail of STUB1 (Fig. 1D). This variant was not identified from the 1514 exome database from Taiwanese healthy controls in the Taiwan Biobank [19]. A further Sanger sequencing of the *STUB1* gene in the remaining 249 patients with molecularly undiagnosed cerebellar ataxia syndrome did not find additional pathogenic variants.

The existence of the heterozygous p.Glu278fs mutation residing in the conserved domain of the CHIP protein encoded by *STUB1* suggests that this rare variant may change the function of CHIP. The acidic residue 278 is conserved from the fruit fly to the human CHIP homologs and is located within the U-box domain (Fig. 1D) [31], the domain responsible for CHIP ubiquitin ligase activity [32]. CHIP not only functions as a ubiquitin ligase but also serves as a co-chaperone through its interactions with HSP70 and HSP90 via its tetratricopeptide repeat (TPR) domain (Fig. 1D). Both the U-box and TPR domains are necessary for CHIP’s ability to control protein quality and attenuate various cellular stress responses [6]. This p.Glu278fs mutation has never before been identified but this residue is very close to a previously reported c.823_824delCT (p.L275Dfs*16) variant in *STUB1* [11]. Taken together, these data demonstrate that this novel heterozygous frameshift variant located in the conserved C-terminal tail of CHIP and segregating with the disease may impact the function of CHIP, which may impair protein homeostasis and contribute to cellular dysfunction.

### Expression of the CHIP p.Glu278fs mutant causes neuronal α-synuclein aggregation

**Fig. 2:**
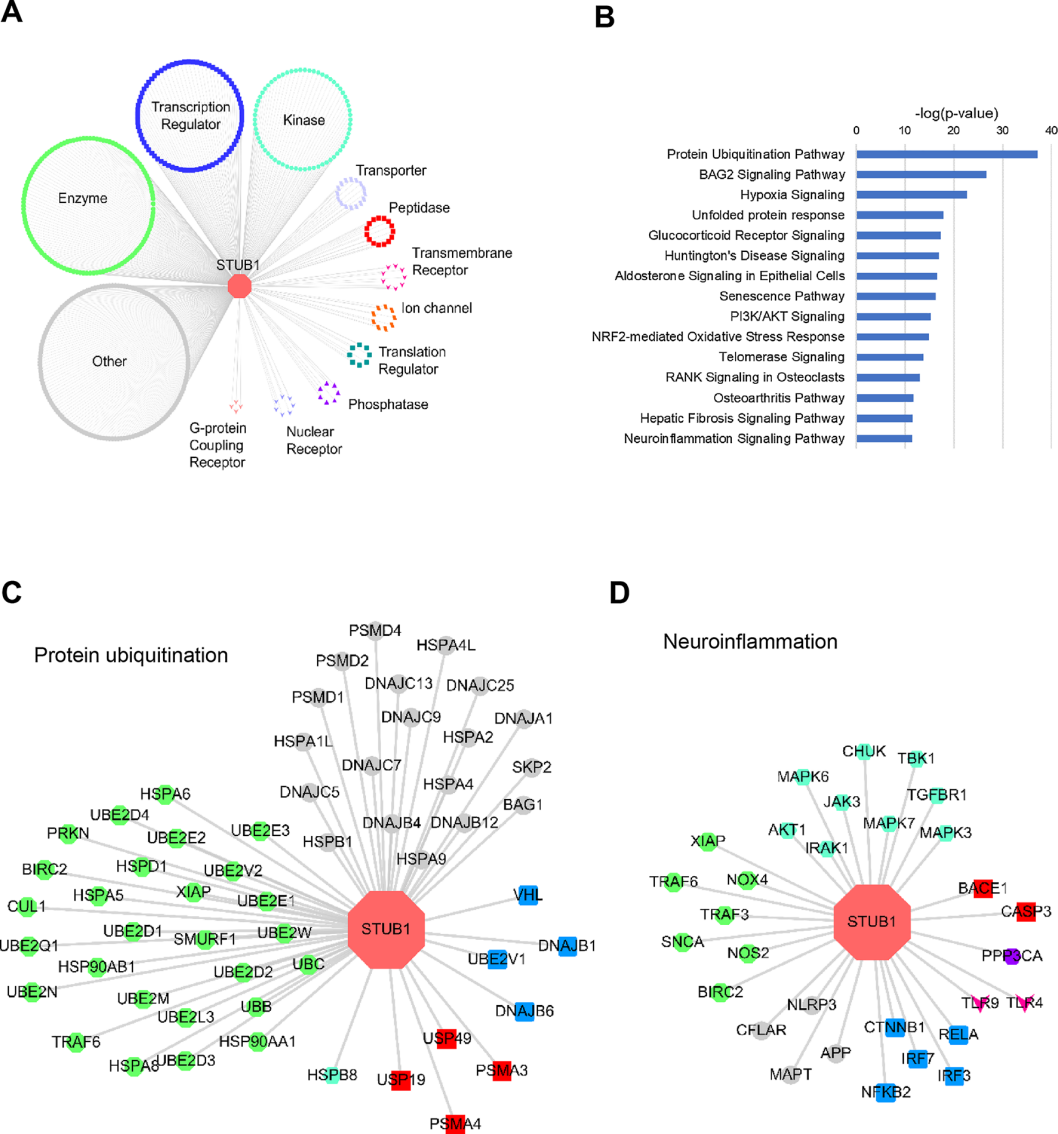
STUB1 interactome analysis. **A** STUB1-interacting proteins retrieved from the IPA database. Node shape and color denote the different molecular types. **B** Top 15 significant enriched pathways of STUB1-interacting proteins. **C** and **D** Subnetworks of STUB1-interacting proteins involved in protein ubiquitination and neuroinflammation pathways

To investigate the potential effect of the identified novel frameshift *STUB1* variant in the function of CHIP and its cellular consequences, we constructed a *STUB1* interactome using the IPA Tool [27]. There were 508 CHIP-interacting proteins retrieved from IPA. Their functions include transcription/translation regulator, kinase/phosphatase, ion channel, transporter, and different kinds of receptors (Fig. 2A). Interestingly, the CHIP-interacting proteins were significantly enriched in pathways of protein ubiquitination, unfolded protein responses, and neuroinflammation, which are all related to the known pathological mechanisms of neurodegenerative diseases (Fig. 2B) [33]. Several interacting proteins are known to be the pathological hallmarks of neurodegenerative diseases, such as tau protein encoded by *MAPT*, α-synuclein encoded by *SNCA*, and ubiquitin conjugating enzyme E2 D1 (UBE2D1) (Fig. 2C and D). Among them, α-synuclein and tau are two of the candidate molecules that may be related to the function of CHIP, as impaired quality control of these two proteins is related to neurodegeneration [34].

**Fig. 3:**
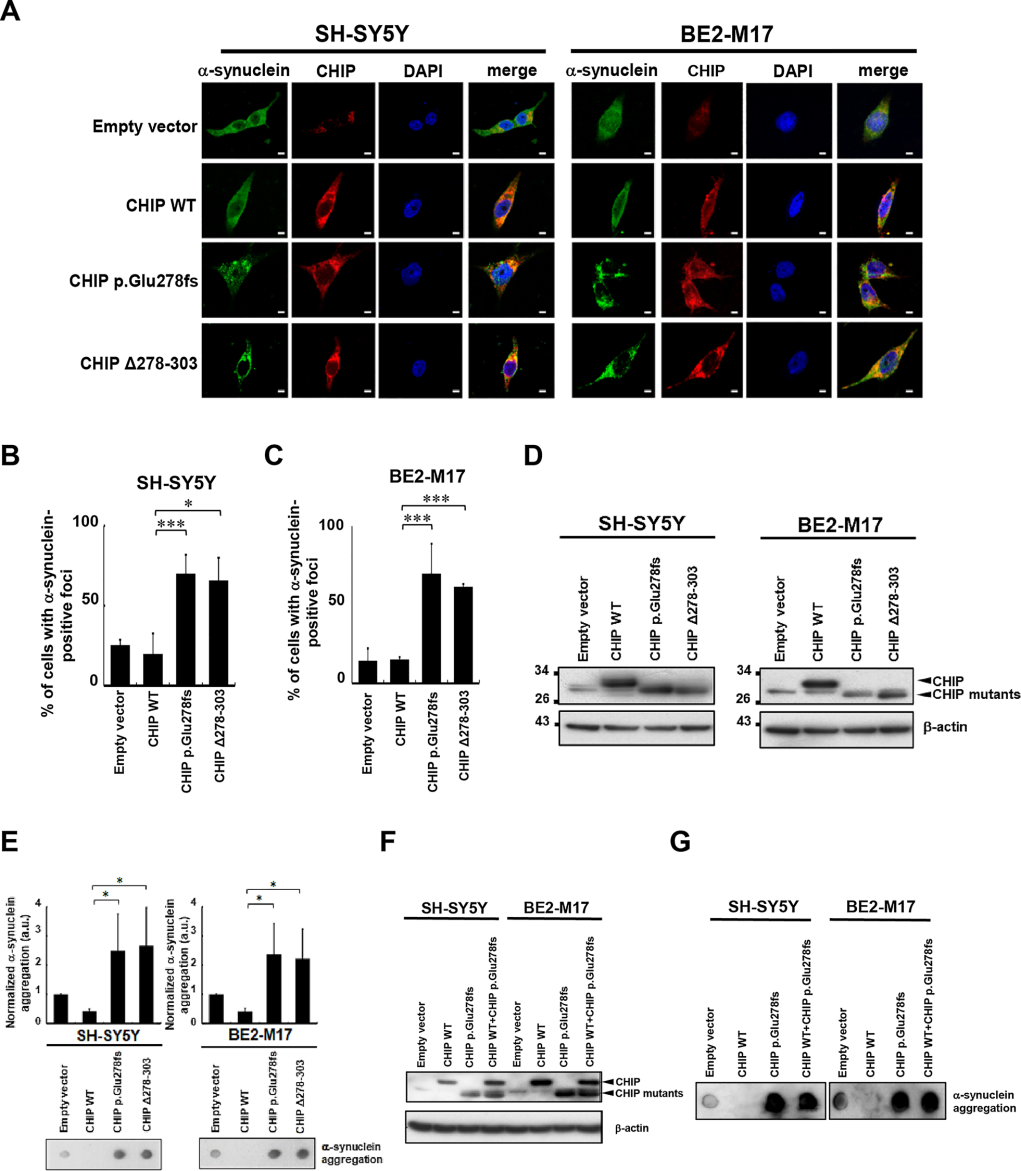
The CHIP mutations promote α-synuclein aggregation in SH-SY5Y and BE2-M17 cells. SH-SY5Y and BE2-M17 cells were transfected for 48 h with WT or mutant CHIP. **A** Confocal Images of SH-SY5Y and BE2-M17 cells following CHIP WT, p.Glu278fs, or CHIP Δ278-303 ectopic expression were captured. Scale bar, 10 μm. **B**, **C** Quantified results in **A** are shown as the percentage of cells with α-synuclein aggregated foci. **D** Ectopic expression of CHIP WT, p.Glu278fs, or Δ278–303 in **A** was examined using Western blot analysis. **E** CHIP mutants increase SDS-insoluble aggregation of α-synuclein in SH-SY5Y and BE2-M17 cells. α-synuclein aggregation was detected by the filter-trap assay in cells transfected with the CHIP WT, p.Glu278fs, or Δ278–303 plasmid. The lysate was diluted in SDS and filtered through nitrocellulose membranes. α-synuclein immunostaining was detected using α-synuclein antibody. A representative image and the densitometry data are shown (a.u., arbitrary unit). The values of α-synuclein aggregation were normalized to the amount of aggregation in the empty vector control (one-way ANOVA, *p < 0.05, ***p < 0.001). **F**, **G** The α-synuclein aggregations detected by a filter trap assay in **F** SH-SY5Y and BE2-M17 cells overexpressing both wild-type and mutant CHIP at the same time **G** were comparable between cells expressing CHIP p.Glu278fs mutant alone and those co-expressing both CHIP WT and p.Glu278fs mutant

α-Synuclein is predicted to be a substrate of the CHIP-associated protein degradation pathway, and α-synuclein-containing protein aggregates lead to neuronal degeneration, which is a pathological feature of many neurodegenerative disorders. Recent evidence suggests that CHIP associates with α-synuclein and reduces the levels of pathological α-synuclein oligomers via both lysosomal and proteasomal pathways [35, 36]. CHIP overexpression enhances the ubiquitination of α-synuclein [24]. As α-synuclein is a substrate of CHIP and CHIP’s E3 activity is sufficient to ubiquitinate α-synuclein [6, 24], we next investigated whether the p.Glu278fs mutation affects CHIP’s function in the clearance of α-synuclein accumulation and aggregation. We detected α-synuclein aggregated foci in CHIP wild-type (WT)- or p.Glu278fs-expressing neuronal-like SH-SY5Y and BE2-M17 cells, using an immunofluorescence assay. Strikingly, the α-synuclein foci were increased in the p.Glu278fs-expressing cells, suggesting that the CHIP p.Glu278fs mutation may increase α-synuclein aggregation (Fig. 3A–C). The p.Glu278fs results in a + 1 shift in the reading frame at residue 278, which adds 8 amino acids (aa) of frameshifted translation before the stop codon. To understand whether the defect in CHIP p.Glu278fs is from the original C-terminal 26-aa deletion or the additional 8-aa extension, a CHIP Δ278–303 construct was created in which a stop codon was inserted at the aa residue 278 (Fig. 3D). Notably, the α-synuclein aggregations were also increased in the CHIP Δ278–303-expressing cells (Fig. 3A, lower panel, statistics in Fig. 3B, C). These results suggest that the C-terminal truncation of CHIP inhibits its clearance of α-synuclein aggregates.

In addition to the microscopic visualization of α-synuclein aggregates, we further used a filter-trap assay to measure the amount of SDS-insoluble α-synuclein inclusions [37, 38]. Both the CHIP p.Glu278fs and CHIP Δ278–303 mutations increased the amounts of SDS-insoluble aggregates of α-synuclein in SH-SY5Y and BE2-M17 cells (Fig. 3E). In contrast, ectopic expression of the wild-type CHIP decreased SDS-insoluble α-synuclein aggregates. To further determine the observed effect of the CHIP p.Glu278fs mutation on α-synuclein aggregations is through the dominant-negative or haploinsufficiency effect, we performed the filter trap assay in SH-SY5Y and BE2-M17 cells co-expressing both wild-type and mutant CHIP at the same time (Fig. 3F). The α-synuclein aggregations were comparable between cells expressing CHIP p.Glu278fs mutant alone and those co-expressing CHIP WT and CHIP p.Glu278fs mutant (Fig. 3G). These results suggest that the CHIP p.Glu278fs mutation causes α-synuclein aggregations through a potential dominant-negative effect.

### The CHIP p.Glu278fs mutation triggers tau aggregation

**Fig. 4:**
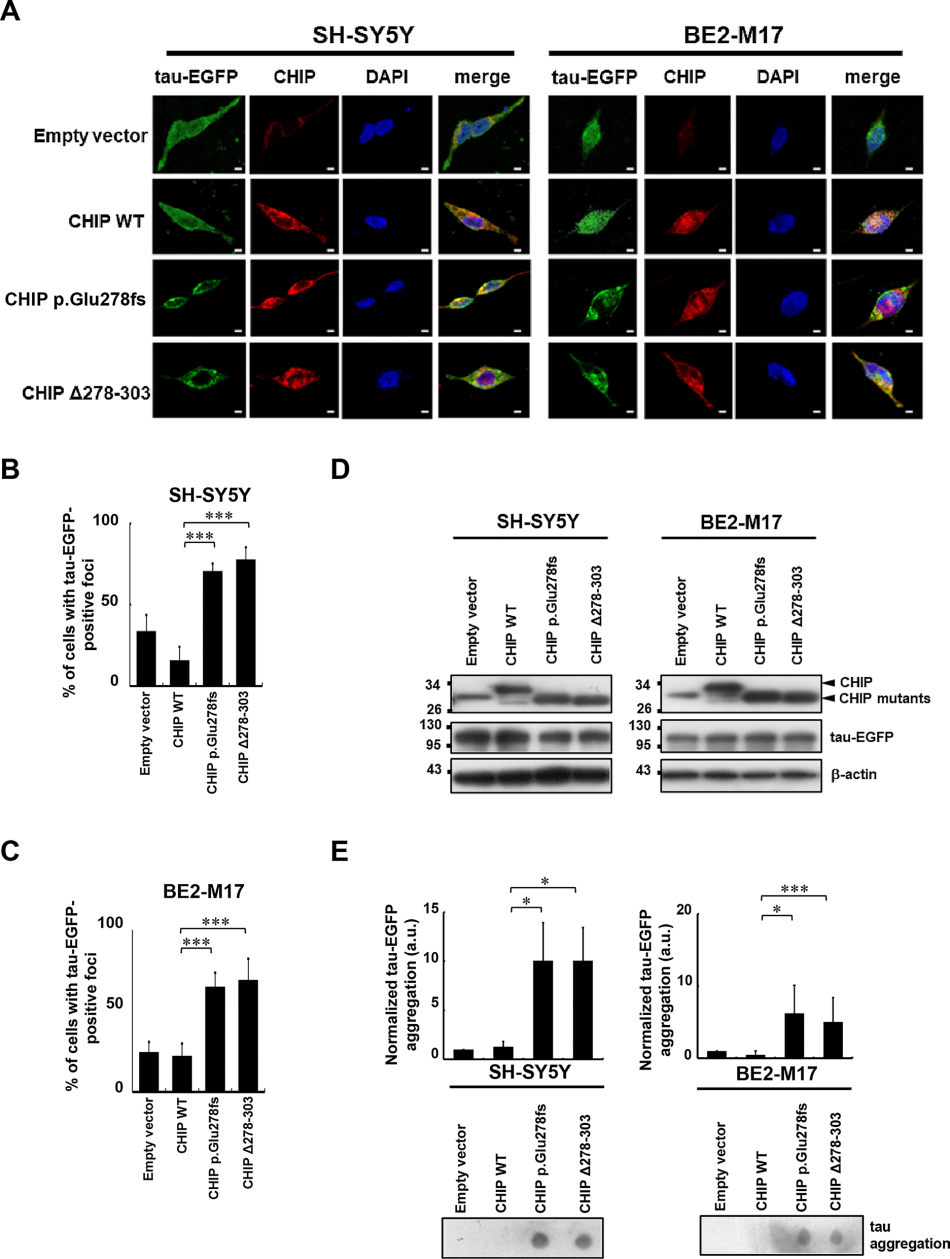
The CHIP mutations cause tau aggregation. **A** After 48-h transfection, SH-SY5Y and BE2-M17 cells were paraformaldehyde-fixed and DAPI (blue) was used to stain the nuclear DNA. The images (× 630) were acquired using a Zeiss LSM780 laser scanning fluorescence confocal microscope. Scale bar, 10 μm. **B**, **C** Quantified results in **A** are shown as the percentage of tau aggregated foci in SH-SY5Y and BE2-M17 cells. **D** Ectopic expression of CHIP WT, p.Glu278fs, or Δ278–303 in **A** was examined using Western blot analysis. **E** CHIP mutants increase SDS-insoluble aggregation of tau in SH-SY5Y and BE2-M17 cells. Tau aggregation was detected by the filter-trap assay in cells transfected with the CHIP WT, p.Glu278fs, or Δ278–303 plasmid. The lysate was diluted in SDS and filtered through nitrocellulose membranes. Tau immunostaining was detected using the tau antibody. A representative image and the densitometry data are shown (a.u., arbitrary unit). The values of tau aggregation were normalized to the amount of aggregation in the empty vector control (one-way ANOVA, *p < 0.05, ***p < 0.001)

Tau, a group of neuronal microtubule-associated proteins, plays a key role in the pathology of many neurodegenerative disorders. Several previous studies have reported that CHIP interacts directly with HSP70/90 to induce the ubiquitination of tau [39–41]. Moreover, overexpression of CHIP protects tau from aggregation, while a deficiency of CHIP may induce an increase in insoluble tau [42]. To ascertain whether the CHIP p.Glu278fs mutation is associated with tau aggregation, immunofluorescent staining was performed in WT- and CHIP mutant-expressing SH-SY5Y and BE2-M17 cells. Compared with cells transfected with the empty vector or WT CHIP, cells expressing either CHIP mutant (p.Glu278fs and Δ278–303) exhibited tau-EGFP aggregation (Fig. 4A–C). We further examined the formation of SDS-insoluble inclusions of tau [43] in CHIP mutant-expressing cells. Consistently, a significant increase in the amount of tau aggregates was seen in CHIP mutant-expressing cells (Fig. 4E). Together, these results suggest that the CHIP p.Glu278fs mutation leads to tau aggregation.

### The CHIP p.Glu278fs mutation impairs its interaction with E2 ubiquitin ligase UbE2D1

**Fig. 5:**
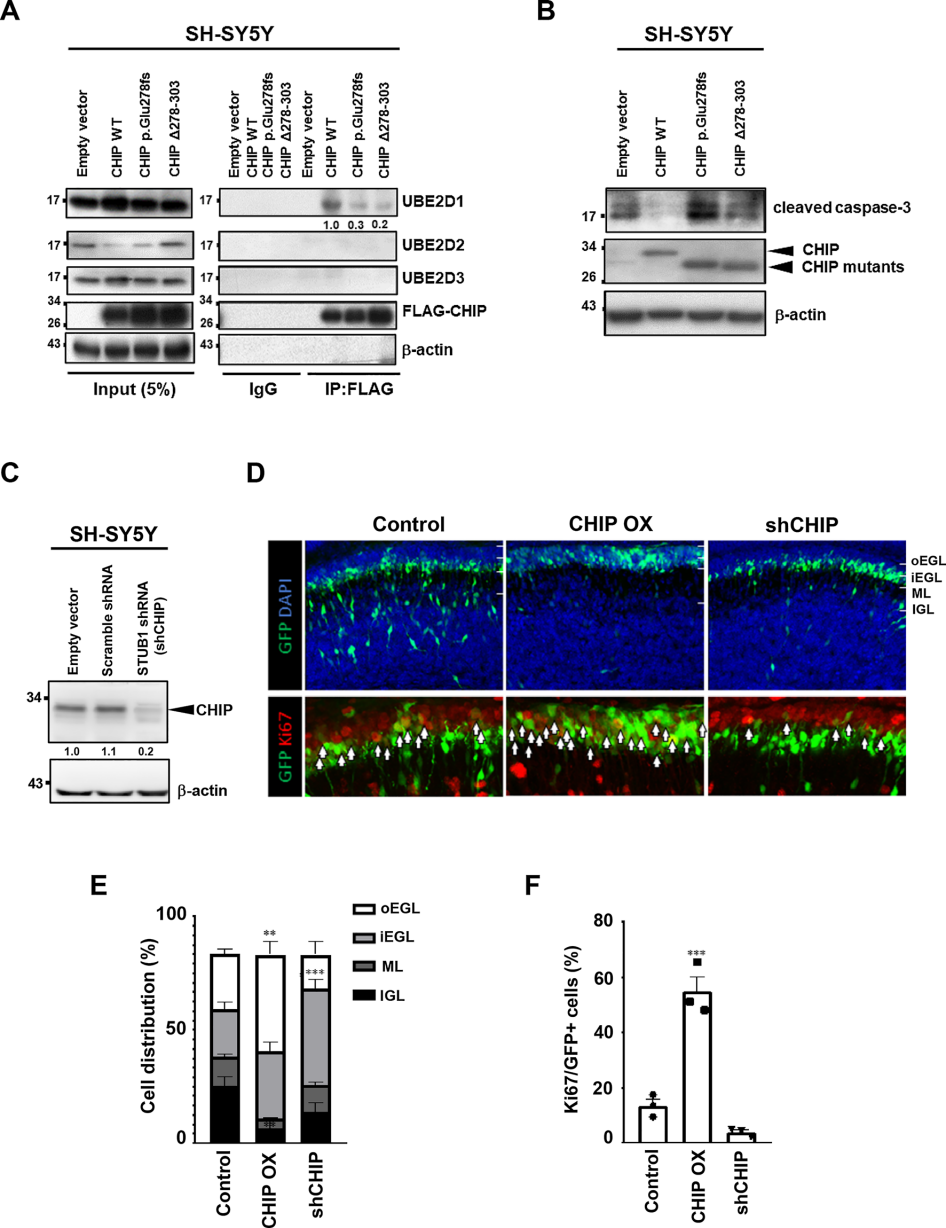
The CHIP mutations abolish the interaction between E2 ubiquitin ligase and CHIP, enhance caspase-3 cleavage and alter cerebellum development. **A** SH-SY5Y cells were transfected with the CHIP WT, p.Glu278fs, or Δ278–303 plasmid for 48 h. Immunoprecipitations were performed using an anti-FLAG antibody. Immunoprecipitates were sequentially probed with anti-UBE2D1, anti-UBE2D2, anti-UBE2D3, and anti-FLAG antibodies. Five percent of lysates used for immunoprecipitation were loaded as the inputs and probed with anti-UBE2D1, anti-UBE2D2, anti-UBE2D3, and anti-FLAG antibodies. IgG served as an IP negative control, and β-actin as a loading control. **B** SH-SY5Y cells were transfected with the CHIP WT, p.Glu278fs, or Δ278–303 plasmid for 48 h and subjected to Western blot analysis. Cleaved caspase-3 was detected. β-actin served as a loading control. **C** SH-SY5Y cells were transfected with the plko.1-puro empty vector, plko.1-puro scramble shRNA and plko.1-puro *STUB1* shRNA plasmid for 48 h and subjected to Western blot analysis. CHIP was detected to examine the knockdown efficiency of plko.1-puro *STUB1* shRNA. β-actin served as a loading control. (D) Mouse cerebellum was electroporated with CHIP cDNA or shCHIP along with GFP at P6 and dissected 2 days later. The upper panel shows the distribution of electroporated GFP + GNPs (green). The lower panel shows the staining of the cell cycle marker Ki67 (red). Brain slices were stained with the DNA dye, DAPI (blue). Arrows: GFP + , Ki67 + cells. **E** Bar graph showing the distribution of GFP + cells in different layers. In the control cerebellum, about half the GNPs migrated from the EGL to the ML and IGL. Overexpression of CHIP arrested cells mostly in the oEGL, while knockdown of CHIP arrested cells mostly in the iEGL. **F** Bar graph showing the percentage of Ki67 + cells among all GFP + cells. CHIP overexpression leads to a dramatic increase in the percentage of Ki67 + cells. (***p < 0.001, **p < 0.002, n = 3 animals, one-way ANOVA.)

The CHIP-mediated ubiquitin transfer cascade requires the sequential E1 activating, E2 conjugating, and E3 ligating enzymes. The CHIP U-box domain can bind both E2-Ub conjugates and substrates to facilitate the transfer of the ubiquitin molecule [44–46]. Given that the CHIP frameshift mutation identified in our patients resides within the ubiquitin ligase region (U-box), we hypothesized that the p.Glu278fs mutation disrupts the E2–U box interaction. To test the effect of the p.Glu278 frameshift mutation on CHIP’s ubiquitin ligase activity, we first screened for E2 candidates that physically interact with CHIP from the NCBI interaction [47, 48] and the STRING databases (https://string-db.org/) [49]. The predicted E2 candidate was selected based on the intersection of two data frames (Additional file 1: Table S4). From the analyses, UbE2D1, UbE2D2, and UbE2D3 are predicted potential targets. We subsequently tested the interaction between CHIP and these E2 candidates using the co-IP assay. Notably, CHIP interacted with UbE2D1, but not UbE2D2 and UbE2D3 (Fig. 5A), and the CHIP mutations inhibited this interaction. Together, these data suggest that the CHIP p.Glu278fs mutation may impair its interaction with the E2 ubiquitin ligase UbE2D1.

### CHIP mutations enhance caspase-3 cleavage

Since caspase-3, a marker of cellular apoptosis, is increased in peripheral tissues in CHIP^–/–^ mice [50, 51], we speculated that both the CHIP p.Glu278fs and CHIP Δ278-303 mutations may cause caspase-3 cleavage. To test this hypothesis, SH-SY5Y cells were transfected with CHIP WT or mutant plasmids (p.Glu278fs and Δ278–303). Ectopic expression of the CHIP mutants increased the cleavage product of caspase-3 (cleaved caspase-3) compared with its level in cells transfected with CHIP WT (Fig. 5B). These results suggest that the mutant CHIP proteins may cause cellular apoptosis, which would further promote the progression of cerebellar ataxia.

### p.Glu278fs mutation does not alter the dimerization capability of CHIP

CHIP is a multi-domain protein with a well-defined architecture. The highly conserved glutamate residue at aa 278 locates within the U-box domain (Fig. 1D). Since the U-box may modulate the homodimerization of CHIP and homodimerization is required for CHIP’s ubiquitin ligase activity [1, 2], we next examined whether the Glu 278 frameshift mutation influences the dimerization property of CHIP. The Myc-tagged wild type CHIP (CHIP WT-Myc) and FLAG-tagged WT or mutant CHIP (FLAG-CHIP p.Glu278fs) were co-transfected into SH-SY5Y and BE2-M17 cells. The co-IP experiments were performed using either an anti-FLAG antibody or an anti-Myc antibody. Compared to that of the wild-type CHIP, co-IP of the CHIP mutants did not significantly alter the physical interaction with wild-type CHIP in both SH-SY5Y and BE2-M17 cells (Additional file 2: Fig. S1). These results suggest that the p.Glu278fs mutation does not change the dimerization capability of CHIP.

### Manipulation of CHIP expression in the developing cerebellum leads to altered differentiation and migration of cerebellar granule neuron progenitors

To investigate the potential roles of CHIP in the cerebellum in vivo, we knocked down and overexpressed CHIP in cerebellar granule neuron progenitors (GNPs) by electroporation of *STUB1* shRNA and cDNA (Fig. 5C), respectively. In control mouse brains electroporated with green fluorescent protein (GFP), about half the GNPs started to differentiate into granule neurons and migrated deep toward the internal granule layer (IGL). Overexpression of CHIP led to an accumulation of GNPs in the outer external granule layer (oEGL) (Fig. 5D, E). By staining the cell-cycle marker Ki67, we found that most CHIP-overexpressing GNPs were still in the cell cycle (Fig. 5D–F). In contrast, knockdown of CHIP delayed GNP migration in the inner EGL (iEGL) (Fig. 5D, E). Interestingly, these cells were negative for Ki67, indicating that they had exited the cell cycle (Fig. 5F). These results suggest that variation in CHIP expression levels plays a critical role in GNP proliferation and migration during cerebellar development, reinforcing the role of *STUB1* mutations in SCA48.

## Discussion

In this study, we identified a novel heterozygous frameshift *STUB1* mutation, c.832del (p.Glu278fs), in an autosomal-dominant three-generational ataxia family among a cohort of patients manifesting cerebellar ataxia syndrome. The affected family members of the index family presented with slowly progressive ataxia and cognitive decline, and their brain MRIs feature diffuse pan-cerebellar atrophy without the involvement of the brainstem or basal ganglia, fitting the diagnosis of SCA48 [11]. Further in vitro experiments demonstrated that this novel heterozygous *STUB1* mutation compromises CHIP’s activity by impairing its interaction with the E2 ubiquitin ligase, UbE2D1, leading to disturbed protein homeostasis in neurons. The in vivo study revealed that the expression level of CHIP is critical for the differentiation and migration of cerebellar neuronal progenitor cells during cerebellar development. Our findings provide clinical, genetic, and functional evidence implicating that the novel heterozygous *STUB1* frameshift mutation at the highly conserved U-box domain of CHIP impairs cerebellar neuronal development and also promotes neuronal apoptosis due to impairs protein homeostasis in neurons through a dominant-negative effect.

The homozygous and compound heterozygous *STUB1* mutations were originally identified in autosomal recessive families with SCAR16 [52, 53]. Recently, single heterozygous *STUB1* mutations were identified in autosomal dominant familial ataxia, [9, 11, 52–55] indicating heterozygous *STUB1* mutation as a cause of autosomal dominant SCA48. The single heterozygous *STUB1*c.832del (p.Glu278fs) mutation identified in our index family is predicted to result in a frameshift mutation affecting the highly conserved U-box domain, which location is similar to those of previously reported variants causing SCA48 [9, 11]. Interestingly, the same phenomena occur with the *SPTBN2* gene, encoding spectrin, in which dominant and recessive mutations cause both SCA5 and SCAR15 [54]. SCAR16 is a predominantly ataxia disorder combined with other features, including seizure, myoclonus, lower limb spasticity, peripheral sensory neuropathy, and hypogonadism, with a typical onset in the second decade [9]. Instead, patients with SCA48 present with relatively late-onset ataxia combined with variable degrees of cognitive decline, mostly dysexecutive, or mood disorders [11, 52, 53, 55]. Heterozygous *STUB1* mutation carriers of SCA48 showed significantly later onset and a less severe expressivity of multi-system neurodegenerative presentations than those with compound heterozygous or homozygous *STUB1* variants in SCAR16. This suggests that the underlying mechanism of SCA48 may be haploinsufficiency. The reported clinical phenotypes of SCA48 are in line with our observations that the main features of our index family members were ataxia with mild cognitive decline and did not have pyramidal signs or movement disorders. The median age of onset of symptoms was 30 years in our patients, slightly younger than that reported in a cohort of European patients with SCA48 (mean onset age 42.4 years) but still older than that of patients with biallelic mutations in *STUB1* in SCAR16 (mean onset age 19.3 years) [55]. This difference may come from different ethnic backgrounds or other modifier genetic effects on the clinical presentation and onset age of SCA48.

**Fig. 6:**
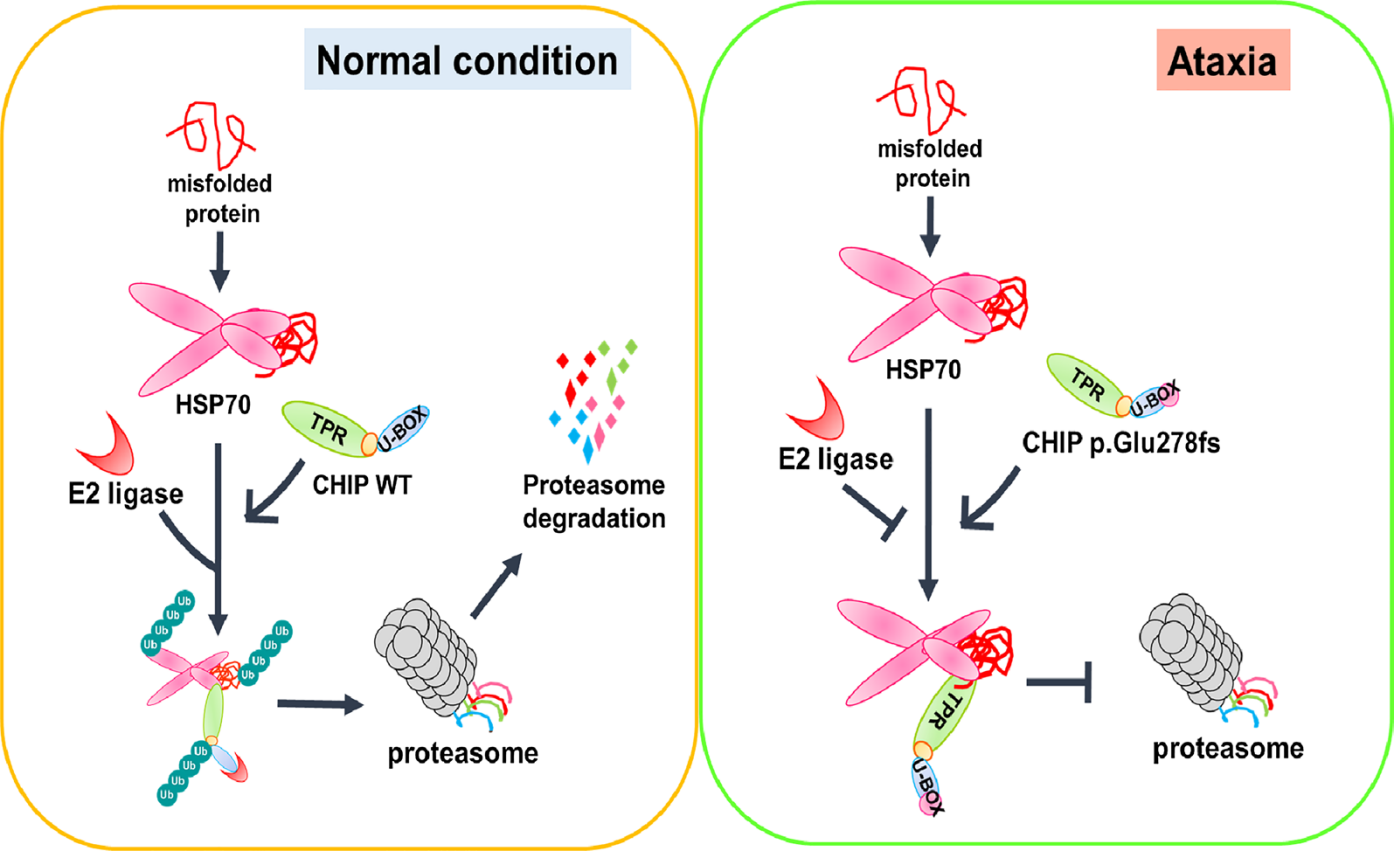
The CHIP p.Glu278fs mutation may impair α-synuclein and tau degradation by disrupting the E2-U-box interaction, thereby promoting ataxia progression. CHIP targets misfolded proteins for proteasome degradation. Under normal conditions, CHIP binds to E2 ligase to form an HSP70 chaperone complex and initiate ubiquitination of the misfolded protein. Under the ataxia condition, the interaction between CHIP and its specific E2 ligase is inhibited, which disrupts CHIP’s E3 ligase activity

Ubiquitin–proteasome system (UPS) dysfunction is associated with neuronal protein aggregates in many neurodegenerative disorders, and genetic mutations and risk alleles in the genes involved in the UPS pathway have been reported in neurodegenerative diseases [44–46]. CHIP, encoded by *STUB1*, is an E3 ubiquitin ligase that selectively directs ubiquitin attachment to specific substrates and plays pivotal roles in protein ubiquitination and protein quality control [6, 56]. CHIP regulates the behaviors of cellular proteins, from their activation, trafficking, subcellular distribution, and interaction with other proteins to their final degradation (Fig. 6). CHIP protein harbors three domains: an N-terminal TPR domain, a highly charged middle coiled-coil domain, and a carboxyl-terminal U-box domain. The TPR domain serves as a protein–protein interaction domain thought to mediate interactions with heat shock proteins, while the U-box domain acts as a ubiquitin ligase. CHIP contains essential N-terminal TPR and C-terminal U-box domains interacting with HSP70/HSP90 and E2, respectively. The U-box domain in CHIP is responsible for the ubiquitination of unfolded proteins destined for proteasomal degradation [56]. A variety of heterozygous missense or deletion variants in *STUB1* contributing to SCA48 have been reported to be located throughout the coding sequence, without evidence of any genotype–phenotype correlation in the severity of cerebellar ataxia [11, 52, 55]. Our identified novel frameshift mutation, which impairs the interaction of CHIP with E2 ubiquitin ligase, extends the mutation spectrum of SCA48. More importantly, our findings provide functional evidence in support of an important role for the C-terminus of CHIP in E2 ligase interaction and clearance of protein aggregates, specifically those of α-synuclein and tau. Notably, our findings from in vitro experiments that tau aggregates were observed in *STUB1* mutant neuronal cells are in line with recent studies showing increased neuronal tau protein aggregation in the post-mortem brain tissues of patients with SCA48 [53, 55]. Furthermore, the in vivo study demonstrated that CHIP expression level affects the neuronal progenitor cells’ differentiation and migration in developing cerebellum. Based on the clinical observations that heterozygous *STUB1* mutation carriers of SCA48 have less severe phenotypes than those with compound heterozygous or homozygous *STUB1* variant carriers of SCAR16, haploinsufficiency is one of the possible mechanisms of SCA48 pathogenesis. Our identified heterozygous frameshift *STUB1* mutation reduces the expression of normal CHIP protein. This may affect cerebellar development and, together with neuronal protein aggregates, may promote cerebellar dysfunction in SCA48. Our observations provide a possible mechanism by which *STUB1* mutations that impair CHIP function lead to altered protein quality control in neurons and consequent neuronal degeneration, and offer a potential target for future mechanism-based therapy for SCA48.

Our study has several limitations. First, the number of familial cases, in either autosomal-dominant or autosomal-recessive pedigrees, was limited. A recent study used Sanger sequencing to explore *STUB1* mutations in 512 Taiwanese families with cerebellar ataxia. It revealed compound heterozygous mutations in 2 families, accounting for 0.4% of the studied cohort, but no single heterozygous mutations in autosomal-dominant families, suggesting either SCAR16 or SCA48 are uncommon forms of familial ataxia in our population [57]. Future genetic screening of *STUB1* in a large cohort of ataxia families in different ethnicities is needed to evaluate the frequency of SCAR16 or SCA48 in patients with familial ataxia syndrome. Furthermore, we assessed cognitive function using complete neuropsychological tests in only two affected members of the index family. A detailed neuropsychological test evaluating individual cognitive domains and a long-term follow-up of cognitive and ataxia motor function are warranted for further assessing the correlation between cerebellar dysfunction and cognitive decline in patients with SCA48. Finally, given that manipulation of CHIP expression can lead to altered differentiation and migration of cerebellar GNPs in vivo, future studies with the CRISPR/Cas9 technology-generated *STUB1* mutant mice may further confirm and explore the *STUB1* mutant-mediated pathogenic effects in the central nervous system.

## Conclusions

In conclusion, our study provides functional evidence linking a novel heterozygous frameshift mutation in the *STUB1* gene to the development of SCA48. Our findings broaden the known mutation spectrum of SCA48 and further stress the importance of CHIP activity in neuronal protein homeostasis and cerebellar functions.

## Supplementary Information

**Additional file 1: Figure S1.** p.Glu278fs mutation does not alter the dimerization property of CHIP. SH-SY5Y and BE2-M17 cells were transfected with CHIP wild-type (WT)-Myc and FLAG-tagged WT or mutant CHIP, including FLAG-CHIP WT, FLAG-CHIP p.Glu278fs, or FALG-CHIP Δ278-303 plasmid for 48 h. Immunoprecipitations were performed using an anti-FLAG antibody or an anti-Myc antibody. Immunoprecipitates were sequentially probed with anti-Myc (A) or anti-FLAG (B) antibodies in two different types of neuronal cell lines. Five percent of lysates used for immunoprecipitation were loaded as the inputs. IgG was served as an IP negative control.

**Additional file 2: Table S1.** The ataxia candidate gene (HP:0001251) list that selected from the Human Phenotype Ontology database. **Table S2.** The mapping information of the whole genome sequencing. **Table S3.** Filtering information of the variants identified from the proband (III-2). **Table S4.** The possible candidates of CHIP’s E2 ligase. 

## Data Availability

Source data are provided with this paper. The datasets generated and analyzed during the current study are available from the corresponding author on reasonable request.
