# Supplementary material for: Clinical and functional characterization of a novel STUB1 frameshift mutation in autosomal dominant spinocerebellar ataxia type 48 (SCA48)
Source: J Biomed Sci. 2021 Sep 26;28:65. doi: 10.1186/s12929-021-00763-1 (PMC8466936; doi:10.1186/s12929-021-00763-1)
Supplement: Supplementary file 1 — Additional file 1: Figure S1. p.Glu278fs mutation does not alter the dimerization property of CHIP. SH-SY5Y and BE2-M17 cells were transfected with CHIP wild-type (WT)-Myc and FLAG-tagged WT or mutant CHIP, including FLAG-CHIP WT, FLAG-CHIP p.Glu278fs, or FALG-CHIP Δ278-303 plasmid for 48 h. Immunoprecipitations were performed using an anti-FLAG antibody or an anti-Myc antibody. Immunoprecipitates were sequentially probed with anti-Myc (A) or anti-FLAG (B) antibodies in two different types of neuronal cell lines. Five percent of lysates used for immunoprecipitation were loaded as the inputs. IgG was served as an IP negative control. [file 12929_2021_763_MOESM1_ESM.docx]

**Supplementary Table 1: The ataxia candidate gene (HP:0001251) list that selected from the Human Phenotype Ontology database.**

| ID | Gene symbol | ID | Gene symbol | ID | Gene symbol |
| --- | --- | --- | --- | --- | --- |
| 1 | AAAS | 37 | ANOS1 | 73 | ATXN10 |
| 2 | AARS1 | 38 | AP1S2 | 74 | ATXN2 |
| 3 | AARS2 | 39 | AP2M1 | 75 | ATXN3 |
| 4 | ABCA5 | 40 | AP3B2 | 76 | ATXN7 |
| 5 | ABCA7 | 41 | AP5Z1 | 77 | ATXN8 |
| 6 | ABCB7 | 42 | APC | 78 | ATXN8OS |
| 7 | ABCC8 | 43 | APOB | 79 | AUH |
| 8 | ABCD1 | 44 | APP | 80 | B4GALNT1 |
| 9 | ABHD12 | 45 | APTX | 81 | B9D1 |
| 10 | ABHD5 | 46 | ARCN1 | 82 | BBS1 |
| 11 | ACADM | 47 | ARL13B | 83 | BCKDHA |
| 12 | ACAT1 | 48 | ARL3 | 84 | BCKDHB |
| 13 | ACD | 49 | ARMC9 | 85 | BCS1L |
| 14 | ACO2 | 50 | ARSA | 86 | BEAN1 |
| 15 | ACOX2 | 51 | ARSG | 87 | BMP15 |
| 16 | ACTL6B | 52 | ARV1 | 88 | BNC1 |
| 17 | ADA2 | 53 | ARX | 89 | BOLA3 |
| 18 | ADAR | 54 | ASL | 90 | BRAT1 |
| 19 | ADGRG1 | 55 | ASS1 | 91 | BSCL2 |
| 20 | ADGRV1 | 56 | ATAD1 | 92 | BTD |
| 21 | ADPRS | 57 | ATAD3A | 93 | C12orf4 |
| 22 | ADSL | 58 | ATCAY | 94 | C12orf65 |
| 23 | AFG3L2 | 59 | ATG5 | 95 | C19orf12 |
| 24 | AGRN | 60 | ATM | 96 | C4A |
| 25 | AGTPBP1 | 61 | ATN1 | 97 | C9orf72 |
| 26 | AHDC1 | 62 | ATP13A2 | 98 | CA8 |
| 27 | AHI1 | 63 | ATP1A2 | 99 | CACNA1A |
| 28 | AIFM1 | 64 | ATP1A3 | 100 | CACNA1B |
| 29 | AKT1 | 65 | ATP2B3 | 101 | CACNA1G |
| 30 | ALDH18A1 | 66 | ATP6 | 102 | CACNA2D2 |
| 31 | ALDH5A1 | 67 | ATP6AP2 | 103 | CACNB4 |
| 32 | ALG11 | 68 | ATP6V0A2 | 104 | CAMTA1 |
| 33 | ALG6 | 69 | ATP6V1A | 105 | CAPN1 |
| 34 | AMACR | 70 | ATP8A2 | 106 | CASK |
| 35 | ANK1 | 71 | ATPAF2 | 107 | CAV1 |
| 36 | ANO10 | 72 | ATXN1 | 108 | CC2D2A |
| 109 | CCDC141 | 149 | COX1 | 189 | DHFR |
| 110 | CCDC28B | 150 | COX10 | 190 | DHX30 |
| 111 | CCDC88C | 151 | COX14 | 191 | DKC1 |
| 112 | CDC42 | 152 | COX15 | 192 | DLAT |
| 113 | CDH23 | 153 | COX2 | 193 | DLD |
| 114 | CDKL5 | 154 | COX20 | 194 | DMXL2 |
| 115 | CEP104 | 155 | COX3 | 195 | DNAJC19 |
| 116 | CEP120 | 156 | COX6B1 | 196 | DNAJC3 |
| 117 | CEP164 | 157 | COX8A | 197 | DNAJC5 |
| 118 | CEP290 | 158 | CP | 198 | DNAJC6 |
| 119 | CEP41 | 159 | CPLANE1 | 199 | DNASE1L3 |
| 120 | CEP78 | 160 | CPS1 | 200 | DNM1 |
| 121 | CFAP43 | 161 | CRAT | 201 | DNM1L |
| 122 | CHAMP1 | 162 | CSF1R | 202 | DNMT1 |
| 123 | CHAT | 163 | CSPP1 | 203 | DOCK3 |
| 124 | CHCHD10 | 164 | CSTB | 204 | DPM1 |
| 125 | CHD2 | 165 | CTBP1 | 205 | DUSP6 |
| 126 | CHD7 | 166 | CTC1 | 206 | DYRK1A |
| 127 | CHP1 | 167 | CTDP1 | 207 | EBF3 |
| 128 | CIB2 | 168 | CTNNA2 | 208 | ECHS1 |
| 129 | CIITA | 169 | CTSD | 209 | EDNRB |
| 130 | CISD2 | 170 | CTSF | 210 | EEF1A2 |
| 131 | CLCN2 | 171 | CUL4B | 211 | EEF2 |
| 132 | CLCN4 | 172 | CWF19L1 | 212 | EIF2S3 |
| 133 | CLN5 | 173 | CXCR4 | 213 | ELN |
| 134 | CLN6 | 174 | CYFIP2 | 214 | ELOVL4 |
| 135 | CLN8 | 175 | CYP27A1 | 215 | ELOVL5 |
| 136 | CLRN1 | 176 | CYP7B1 | 216 | ELP1 |
| 137 | CLTC | 177 | CYTB | 217 | EPB42 |
| 138 | CNKSR2 | 178 | DAB1 | 218 | EPM2A |
| 139 | CNTNAP2 | 179 | DARS2 | 219 | EPRS1 |
| 140 | COA7 | 180 | DBT | 220 | ERCC2 |
| 141 | COA8 | 181 | DCC | 221 | ERCC3 |
| 142 | COG4 | 182 | DCHS1 | 222 | ERCC4 |
| 143 | COG5 | 183 | DCX | 223 | ERCC5 |
| 144 | COG8 | 184 | DDB2 | 224 | ERCC6 |
| 145 | COL13A1 | 185 | DEAF1 | 225 | ERCC8 |
| 146 | COL18A1 | 186 | DEGS1 | 226 | ERMARD |
| 147 | COQ2 | 187 | DGUOK | 227 | ESPN |
| 148 | COQ8A | 188 | DHDDS | 228 | ETHE1 |
| 229 | FA2H | 269 | GDAP2 | 309 | HS6ST1 |
| 230 | FAM149B1 | 270 | GFAP | 310 | HSD17B4 |
| 231 | FAS | 271 | GJA1 | 311 | HTRA1 |
| 232 | FASTKD2 | 272 | GJB1 | 312 | HTT |
| 233 | FAT2 | 273 | GJC2 | 313 | HYLS1 |
| 234 | FAT4 | 274 | GLB1 | 314 | IFT140 |
| 235 | FBXL4 | 275 | GLRA1 | 315 | IL10 |
| 236 | FEZF1 | 276 | GLRB | 316 | IL17RD |
| 237 | FGF12 | 277 | GLRX5 | 317 | IL23R |
| 238 | FGF14 | 278 | GLS | 318 | INPP5E |
| 239 | FGF17 | 279 | GMPPA | 319 | INPP5K |
| 240 | FGF8 | 280 | GMPPB | 320 | INS |
| 241 | FGFR1 | 281 | GNAO1 | 321 | INVS |
| 242 | FLRT3 | 282 | GOSR2 | 322 | IQCB1 |
| 243 | FLVCR1 | 283 | GPAA1 | 323 | IRF2BPL |
| 244 | FMR1 | 284 | GPHN | 324 | ITM2B |
| 245 | FOXI1 | 285 | GPI | 325 | ITPR1 |
| 246 | FOXRED1 | 286 | GRID2 | 326 | KCNA1 |
| 247 | FRMD4A | 287 | GRIN2D | 327 | KCNA2 |
| 248 | FRMPD4 | 288 | GRM1 | 328 | KCNB1 |
| 249 | FSHR | 289 | GRN | 329 | KCNC1 |
| 250 | FTL | 290 | GSN | 330 | KCNC3 |
| 251 | FUS | 291 | GSS | 331 | KCND3 |
| 252 | FXN | 292 | GTF2E2 | 332 | KCNJ10 |
| 253 | GABRA1 | 293 | GTF2H5 | 333 | KCNJ11 |
| 254 | GABRA2 | 294 | GTPBP2 | 334 | KCTD7 |
| 255 | GABRA5 | 295 | HACE1 | 335 | KIAA0556 |
| 256 | GABRB1 | 296 | HARS1 | 336 | KIAA0586 |
| 257 | GABRB2 | 297 | HCN1 | 337 | KIAA0753 |
| 258 | GABRB3 | 298 | HEPACAM | 338 | KIF1A |
| 259 | GABRD | 299 | HERC1 | 339 | KIF1B |
| 260 | GABRG2 | 300 | HESX1 | 340 | KIF1C |
| 261 | GALT | 301 | HEXB | 341 | KIF5A |
| 262 | GAMT | 302 | HIBCH | 342 | KIF7 |
| 263 | GBA | 303 | HIKESHI | 343 | KISS1R |
| 264 | GBA2 | 304 | HLA-B | 344 | KIT |
| 265 | GBE1 | 305 | HLA-DQB1 | 345 | KLLN |
| 266 | GCH1 | 306 | HLCS | 346 | LAGE3 |
| 267 | GCK | 307 | HMGCL | 347 | LAMA1 |
| 268 | GCLC | 308 | HNRNPH2 | 348 | LETM1 |
| 349 | LIPT1 | 389 | MYO7A | 429 | NDUFV1 |
| 350 | LMBRD1 | 390 | MYO9A | 430 | NDUFV2 |
| 351 | LMNB1 | 391 | MYORG | 431 | NECAP1 |
| 352 | LMNB2 | 392 | NADK2 | 432 | NEFL |
| 353 | LNPK | 393 | NAGS | 433 | NEU1 |
| 354 | LRPPRC | 394 | NANS | 434 | NEUROD2 |
| 355 | LYRM7 | 395 | NAT8L | 435 | NEXMIF |
| 356 | LYST | 396 | NAXD | 436 | NF2 |
| 357 | MAB21L1 | 397 | NAXE | 437 | NFASC |
| 358 | MAG | 398 | ND1 | 438 | NFIX |
| 359 | MAN2B1 | 399 | ND2 | 439 | NGLY1 |
| 360 | MAPK8IP3 | 400 | ND4 | 440 | NHLRC1 |
| 361 | MARS2 | 401 | ND4L | 441 | NKX2-1 |
| 362 | MAST1 | 402 | ND5 | 442 | NKX6-2 |
| 363 | MBD5 | 403 | ND6 | 443 | NOL3 |
| 364 | MCOLN1 | 404 | NDUFA1 | 444 | NONO |
| 365 | MECP2 | 405 | NDUFA10 | 445 | NOP56 |
| 366 | MECR | 406 | NDUFA11 | 446 | NOTCH2NLC |
| 367 | MED13L | 407 | NDUFA12 | 447 | NPC1 |
| 368 | MEFV | 408 | NDUFA13 | 448 | NPC2 |
| 369 | MFSD8 | 409 | NDUFA2 | 449 | NPHP1 |
| 370 | MICOS13 | 410 | NDUFA6 | 450 | NPHP3 |
| 371 | MICU1 | 411 | NDUFA9 | 451 | NPHP4 |
| 372 | MKS1 | 412 | NDUFAF1 | 452 | NR5A1 |
| 373 | MLC1 | 413 | NDUFAF2 | 453 | NSD2 |
| 374 | MMADHC | 414 | NDUFAF3 | 454 | NSMF |
| 375 | MME | 415 | NDUFAF4 | 455 | NTRK2 |
| 376 | MPDU1 | 416 | NDUFAF5 | 456 | NUBPL |
| 377 | MPLKIP | 417 | NDUFAF6 | 457 | NUP107 |
| 378 | MPV17 | 418 | NDUFB11 | 458 | NUP214 |
| 379 | MPZ | 419 | NDUFB3 | 459 | NUP62 |
| 380 | MRE11 | 420 | NDUFB8 | 460 | NUS1 |
| 381 | MRPS22 | 421 | NDUFB9 | 461 | OFD1 |
| 382 | MSTO1 | 422 | NDUFS1 | 462 | OGDH |
| 383 | MTFMT | 423 | NDUFS2 | 463 | OPA1 |
| 384 | MTPAP | 424 | NDUFS3 | 464 | OPA3 |
| 385 | MTTP | 425 | NDUFS4 | 465 | OPHN1 |
| 386 | MVK | 426 | NDUFS6 | 466 | OTC |
| 387 | MYD88 | 427 | NDUFS7 | 467 | OTUD6B |
| 388 | MYO5A | 428 | NDUFS8 | 468 | OXR1 |
| 469 | PAFAH1B1 | 509 | PIGG | 549 | PROK2 |
| 470 | PAK1 | 510 | PIGL | 550 | PROKR2 |
| 471 | PANK2 | 511 | PIGO | 551 | PRPS1 |
| 472 | PARN | 512 | PIGP | 552 | PRRT2 |
| 473 | PARS2 | 513 | PIGQ | 553 | PSAP |
| 474 | PAX6 | 514 | PIGS | 554 | PSEN1 |
| 475 | PCDH15 | 515 | PIGT | 555 | PSEN2 |
| 476 | PCDH19 | 516 | PIGV | 556 | PSMC3IP |
| 477 | PCNA | 517 | PIGW | 557 | PTEN |
| 478 | PDE6D | 518 | PIGY | 558 | PTPN22 |
| 479 | PDE8B | 519 | PIK3CA | 559 | PTRH2 |
| 480 | PDGFB | 520 | PIK3R5 | 560 | PTS |
| 481 | PDGFRB | 521 | PLA2G6 | 561 | PUM1 |
| 482 | PDHA1 | 522 | PLD3 | 562 | PYCR2 |
| 483 | PDHX | 523 | PLP1 | 563 | RAB11B |
| 484 | PDP1 | 524 | PMM2 | 564 | RAD50 |
| 485 | PDX1 | 525 | PMP22 | 565 | RARS1 |
| 486 | PDYN | 526 | PMPCA | 566 | REPS1 |
| 487 | PDZD7 | 527 | PMPCB | 567 | RFC1 |
| 488 | PET100 | 528 | PNKP | 568 | RFT1 |
| 489 | PEX1 | 529 | PNP | 569 | RFX5 |
| 490 | PEX10 | 530 | PNPLA6 | 570 | RFXANK |
| 491 | PEX11B | 531 | PNPLA8 | 571 | RFXAP |
| 492 | PEX12 | 532 | POLG | 572 | RIPK4 |
| 493 | PEX13 | 533 | POLG2 | 573 | RNASEH1 |
| 494 | PEX14 | 534 | POLR1C | 574 | RNASET2 |
| 495 | PEX16 | 535 | POLR3A | 575 | RNF113A |
| 496 | PEX19 | 536 | POLR3B | 576 | RNF168 |
| 497 | PEX2 | 537 | POU3F4 | 577 | RNF216 |
| 498 | PEX26 | 538 | PPP1R15B | 578 | RNR1 |
| 499 | PEX3 | 539 | PPP2R2B | 579 | ROGDI |
| 500 | PEX5 | 540 | PPP2R5D | 580 | RORA |
| 501 | PEX6 | 541 | PPP3CA | 581 | RPGRIP1L |
| 502 | PEX7 | 542 | PPT1 | 582 | RPIA |
| 503 | PGAP2 | 543 | PRDM8 | 583 | RPL10 |
| 504 | PGAP3 | 544 | PRF1 | 584 | RRM2B |
| 505 | PGK1 | 545 | PRICKLE1 | 585 | RTEL1 |
| 506 | PGM3 | 546 | PRKCG | 586 | RTN2 |
| 507 | PHYH | 547 | PRNP | 587 | RTN4IP1 |
| 508 | PIBF1 | 548 | PRODH | 588 | RUBCN |
| 589 | SACS | 629 | SLC25A15 | 669 | SQSTM1 |
| 590 | SAMD9L | 630 | SLC25A22 | 670 | SRD5A3 |
| 591 | SARDH | 631 | SLC25A4 | 671 | ST3GAL5 |
| 592 | SARS1 | 632 | SLC25A42 | 672 | STAT3 |
| 593 | SCARB2 | 633 | SLC25A46 | 673 | STAT4 |
| 594 | SCN1A | 634 | SLC26A4 | 674 | STN1 |
| 595 | SCN1B | 635 | SLC2A1 | 675 | STUB1 |
| 596 | SCN2A | 636 | SLC30A10 | 676 | STX1B |
| 597 | SCN3A | 637 | SLC30A9 | 677 | STXBP1 |
| 598 | SCN8A | 638 | SLC35A1 | 678 | SUCLA2 |
| 599 | SCN9A | 639 | SLC39A4 | 679 | SUCLG1 |
| 600 | SCO1 | 640 | SLC46A1 | 680 | SUFU |
| 601 | SCO2 | 641 | SLC4A1 | 681 | SUMF1 |
| 602 | SCYL1 | 642 | SLC52A2 | 682 | SUOX |
| 603 | SDCCAG8 | 643 | SLC52A3 | 683 | SURF1 |
| 604 | SDHA | 644 | SLC5A7 | 684 | SYNE1 |
| 605 | SDHAF1 | 645 | SLC6A1 | 685 | SYNGAP1 |
| 606 | SDHB | 646 | SLC6A19 | 686 | SYNJ1 |
| 607 | SDHC | 647 | SLC6A5 | 687 | SYT1 |
| 608 | SDHD | 648 | SLC6A8 | 688 | SYT14 |
| 609 | SEC23B | 649 | SLC9A1 | 689 | SYT2 |
| 610 | SEMA3A | 650 | SLC9A6 | 690 | SZT2 |
| 611 | SETX | 651 | SMAD4 | 691 | TACO1 |
| 612 | SFXN4 | 652 | SMARCB1 | 692 | TACR3 |
| 613 | SGPL1 | 653 | SMPD1 | 693 | TAF1 |
| 614 | SIK1 | 654 | SNAI2 | 694 | TANGO2 |
| 615 | SIL1 | 655 | SNAP25 | 695 | TARDBP |
| 616 | SLC12A3 | 656 | SNAP29 | 696 | TARS1 |
| 617 | SLC13A3 | 657 | SNORD118 | 697 | TAT |
| 618 | SLC13A5 | 658 | SNX14 | 698 | TBC1D23 |
| 619 | SLC16A2 | 659 | SOX10 | 699 | TBCD |
| 620 | SLC17A5 | 660 | SPART | 700 | TBCE |
| 621 | SLC18A2 | 661 | SPAST | 701 | TBK1 |
| 622 | SLC18A3 | 662 | SPG11 | 702 | TBP |
| 623 | SLC19A2 | 663 | SPG7 | 703 | TCF20 |
| 624 | SLC19A3 | 664 | SPR | 704 | TCF4 |
| 625 | SLC1A2 | 665 | SPRY4 | 705 | TCN2 |
| 626 | SLC1A3 | 666 | SPTA1 | 706 | TCTN1 |
| 627 | SLC20A2 | 667 | SPTB | 707 | TCTN2 |
| 628 | SLC25A1 | 668 | SPTBN2 | 708 | TCTN3 |
| 709 | TDP1 | 749 | TRNS1 | 789 | WDR26 |
| 710 | TDP2 | 750 | TRNS2 | 790 | WDR73 |
| 711 | TECPR2 | 751 | TRNT1 | 791 | WDR81 |
| 712 | TELO2 | 752 | TRNV | 792 | WFS1 |
| 713 | TERC | 753 | TRNW | 793 | WHRN |
| 714 | TERT | 754 | TRPC3 | 794 | WWOX |
| 715 | TGFB1 | 755 | TSFM | 795 | XPA |
| 716 | TGM6 | 756 | TTBK2 | 796 | XPC |
| 717 | TH | 757 | TTC19 | 797 | XRCC1 |
| 718 | TIMMDC1 | 758 | TTPA | 798 | XRCC4 |
| 719 | TINF2 | 759 | TTR | 799 | YME1L1 |
| 720 | TK2 | 760 | TUBA1A | 800 | YWHAG |
| 721 | TMEM106B | 761 | TUBB | 801 | ZBTB11 |
| 722 | TMEM107 | 762 | TUBB2B | 802 | ZFYVE26 |
| 723 | TMEM126B | 763 | TUBB3 | 803 | ZNF142 |
| 724 | TMEM138 | 764 | TUBB4A | 804 | ZNF423 |
| 725 | TMEM216 | 765 | TWNK |  |  |
| 726 | TMEM231 | 766 | UBA5 |  |  |
| 727 | TMEM237 | 767 | UBAP1 |  |  |
| 728 | TMEM240 | 768 | UBE3A |  |  |
| 729 | TMEM67 | 769 | UBTF |  |  |
| 730 | TMEM70 | 770 | UCHL1 |  |  |
| 731 | TOP3A | 771 | UQCRQ |  |  |
| 732 | TPK1 | 772 | UROC1 |  |  |
| 733 | TPP1 | 773 | USH1C |  |  |
| 734 | TPRKB | 774 | USH1G |  |  |
| 735 | TRAF3IP1 | 775 | USH2A |  |  |
| 736 | TRAK1 | 776 | VAMP1 |  |  |
| 737 | TRAPPC11 | 777 | VARS2 |  |  |
| 738 | TRAPPC6B | 778 | VCP |  |  |
| 739 | TREM2 | 779 | VHL |  |  |
| 740 | TREX1 | 780 | VLDLR |  |  |
| 741 | TRIO | 781 | VPS13A |  |  |
| 742 | TRNF | 782 | VPS13D |  |  |
| 743 | TRNI | 783 | VRK1 |  |  |
| 744 | TRNK | 784 | VWA3B |  |  |
| 745 | TRNL1 | 785 | WARS2 |  |  |
| 746 | TRNN | 786 | WASHC5 |  |  |
| 747 | TRNP | 787 | WDR11 |  |  |
| 748 | TRNQ | 788 | WDR19 |  |  |

**Supplementary Table 2. The mapping information of the whole genome sequencing.**

| Sample ID | II-2 | II-3 | III-2 |
| --- | --- | --- | --- |
| Total bases (bp) | 105,752,514,144 | 100,449,068,000 | 101,063,752,007 |
| Average read depth | 35.2 | 33.5 | 33.7 |
| Percentage of >10x coverage (%) | 97.7% | 97.5% | 97.5% |
| Total variants | 4,499,308 | 4,538,154 | 4,477,903 |

**Supplementary Table 3. Filtering information of the variants identified from the proband (III-2)**

| Filtering criteria | Number of variants |
| --- | --- |
| Total variants | 4,538,154 |
| The total variants which its allele frequency less than 1% in the dbSNP 151 and Taiwan biobank database | 278,901 |
| Located in the functional regions (including CDS region, 5’-UTR, 3’-UTR or splicing site) | 1,081 |
| The variant type not belong to benign or likely benign in the ClinVar database | 991 |
| Located in the ataxia candidate gene list | 17 |
| Shared common variants from the proband’s affected mother | 7 |
| Affects the protein function | 4 |
| The inheritance model of the gene has been reported as dominant model in the OMIM database | 2 |

**Supplementary Table 4. The possible candidates of CHIP’s E2 ligase.**

| Interactant | Gene | Source | Description |
| --- | --- | --- | --- |
| BioGRID:113169 | UBE2D1 | BioGRID | Reconstituted Complex |
| BioGRID:113170 | UBE2D2 | BioGRID | Biochemical Activity; Co-purification; Reconstituted Complex |
| BioGRID:113171 | UBE2D3 | BioGRID | Affinity Capture-Western; Reconstituted Complex |
| BioGRID:119641 | UBE2D4 | BioGRID | Reconstituted Complex |
| BioGRID:113172 | UBE2E1 | BioGRID | Reconstituted Complex |
| BioGRID:113173 | UBE2E2 | BioGRID | Reconstituted Complex |
| BioGRID:115740 | UBE2E3 | BioGRID | Reconstituted Complex |
| BioGRID:113180 | UBE2L3 | BioGRID | Biochemical Activity; Reconstituted Complex |
| BioGRID:114504 | UBE2M | BioGRID | Affinity Capture-MS |
| BioGRID:113182 | UBE2N | BioGRID | Affinity Capture-Luminescence; Affinity Capture-MS; Affinity Capture-Western; Reconstituted Complex |
| BioGRID:120732 | UBE2Q1 | BioGRID | Protein-peptide; Reconstituted Complex; Two-hybrid |
| BioGRID:113183 | UBE2V1 | BioGRID | Affinity Capture-MS; Co-fractionation; Reconstituted Complex |
| BioGRID:113184 | UBE2V2 | BioGRID | Co-fractionation; Reconstituted Complex |
| BioGRID:120572 | UBE2W | BioGRID | Biochemical Activity; Reconstituted Complex |
