# Supplementary figures and images for: Clinical and functional characterization of a novel STUB1 frameshift mutation in autosomal dominant spinocerebellar ataxia type 48 (SCA48)
Source: J Biomed Sci. 2021 Sep 26;28:65. doi: 10.1186/s12929-021-00763-1 (PMC8466936; doi:10.1186/s12929-021-00763-1)

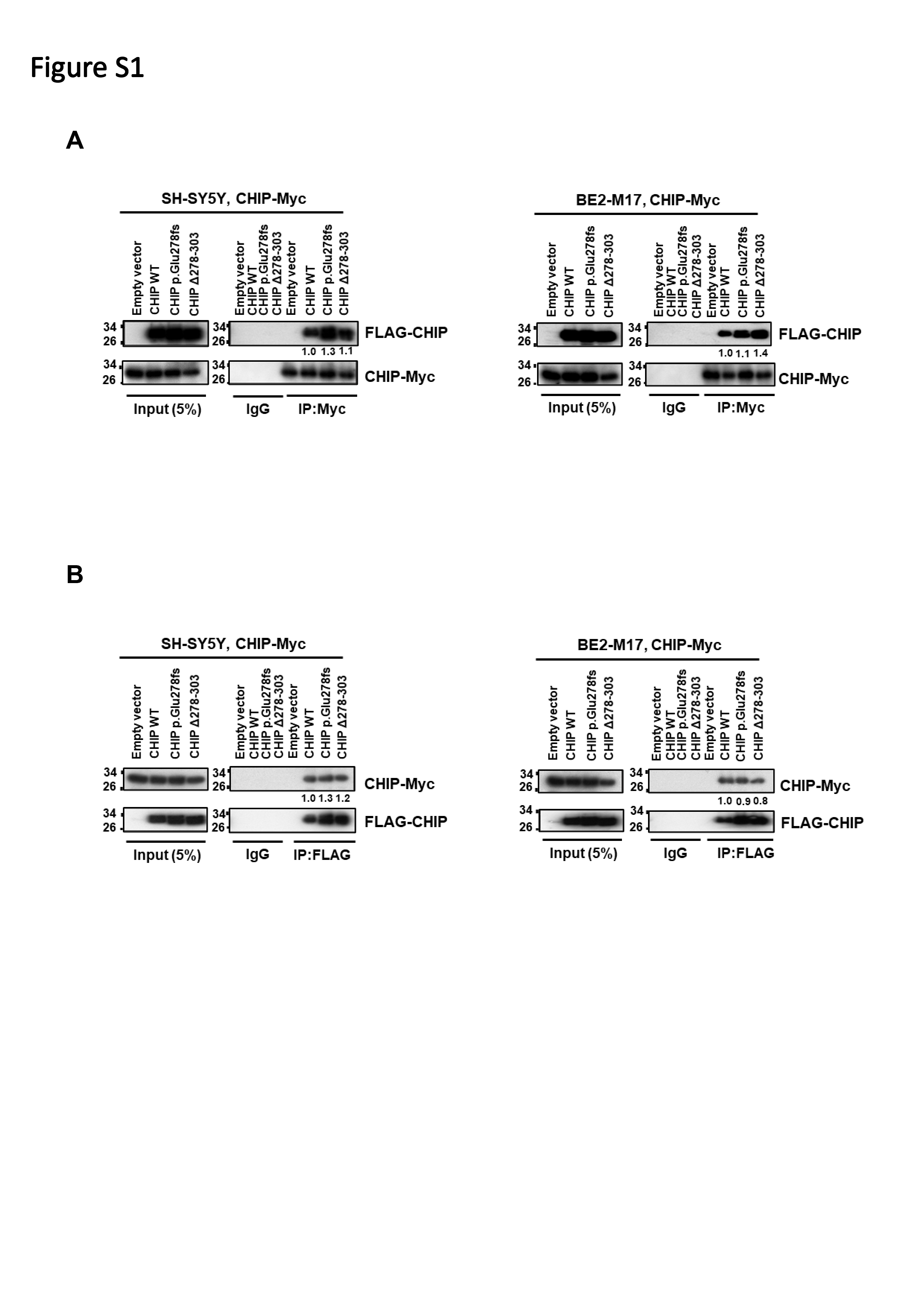

Supplement: Supplementary file 2 — Additional file 2: Table S1. The ataxia candidate gene (HP:0001251) list that selected from the Human Phenotype Ontology database. Table S2. The mapping information of the whole genome sequencing. Table S3. Filtering information of the variants identified from the proband (III-2). Table S4. The possible candidates of CHIP’s E2 ligase. [file 12929_2021_763_MOESM2_ESM.tif]
